# Progress for Journal of Functional Morphology and Kinesiology in 2018

**DOI:** 10.3390/jfmk4010004

**Published:** 2019-01-06

**Authors:** Giuseppe Musumeci

**Affiliations:** Department of Biomedical and Biotechnological Sciences, Human Anatomy and Histology Section, School of Medicine, University of Catania, Via S. Sofia 87, 95123 Catania, Italy; g.musumeci@unict.it

## 1. Looking Back on 2018

The Journal of Functional Morphology and Kinesiology (JFMK, ISSN: 2411-5142), which was firstly released in March 2016 [1], developed greatly in 2018. This journal provides an advanced forum for research studies on functional morphology and kinesiology and the regulatory functions of movement. JFMK meets the growing demand for high-quality, peer-reviewed international journals, supplying easy access, high publicity of Open Access, the Digital Object Identifier (DOI), ORCID, and CrossRef to all researchers. We are indexed in the DOAJ (Directory of Open Access Journals), Scilit (a comprehensive, open-access scholarly database, developed and maintained by MDPI), Google Scholar, and World Health Organization Hinari. Our full texts are archived in CLOCKSS (Digital Archive), e-Helvetica (Swiss National Library Digital Archive), and J-Gate (Informatics India). This year we were indexed by Scopus (Elsevier’s abstract and citation database). We hope we can be included in Web of Science and PubMed in the near future, even if 4 JFMK papers are already indexed on PubMed (https://www.ncbi.nlm.nih.gov/pubmed/?term=jfmk). JFMK is a member of the Committee on Publication Ethics (COPE). To verify the originality of content submitted to our journals, we still use iThenticate to check submissions against previous publications. MDPI works with Publons to provide reviewers with credit for their work and MDPI Scitations Alert to provide our authors information on new publications in their research field. Kinesiology is the study of human movement or physical activity related to many sub-disciplinary areas, such as athletic training, biomechanics, exercise physiology, psychology, leisure, measurement, motor behavior, public health, and sociology.

We are proud to let you know that thanks to your continuous support, Journal of Functional Morphology and Kinesiology has continued to grow in the fields of morphology, kinesiology, movement, biomechanics, sport medicine, and musculoskeletal disorders. It is my pleasure to confirm the progress recorded in 2017 and 2018 [2,3].

Indeed, the number of published manuscripts has jumped from 89 in the 2017 volume to 154 in the 2018 volume, with 16 scientific contributions for each issue, and we rejected in total 34 contributions to maintain the high standards of our journal. The Journal of Functional Morphology and Kinesiology receives more manuscripts than it is able to publish and the decision as to which papers are accepted or rejected is a difficult one. The decision is based on several factors, including originality, experimental design, scientific quality, data interpretation, clarity, and English quality, to maintain the high standards of our journal.

In 2018, the sections of JFMK have been updated to eleven parts: Functional Anatomy; Kinesiology and Biomechanics; Sport Medicine and Nutrition; Motor Control and Rehabilitation; Athletic Training and Human Performance; Gait and Posture; Mechanobiology; Movement and Neurodegenerative Diseases; Musculoskeletal Disorders; Physical Exercise for Health Promotion; and Strength and Conditioning.

In 2018, different special issues were activated thanks to the huge support of our editors. They include the following: “Resistance Training for Performance and Health”, edited by Prof. Dr. Antonio Paoli [4]; “Arrhythmic Events in Sports Medicine and Kinesiology”, edited by Dr. Laura Stefani [5]; “Sarcopenia and Muscle Wasting”, edited by Prof. Dr. Giuseppe Musumeci [6]; “Selected Papers from icSPORTS 2018”, edited by Prof. Dr. Jan Cabri, Prof. Dr. Pedro Pezarat-Correia, and Prof. Dr. João Paulo Vilas-Boas [7]; “Sport Psychology”, edited by Prof. Lídia Serra [8]; “Exercise-Induced Immune Response”, edited by Prof. Francesca Luchetti [9]; "Eccentric Exercise: Adaptations and Applications for Health and Performance", edited by Dr. Michael O. Harris-Love, Dr. Jared M. Gollie, and Dr. Justin Keogh [10].

In 2018, ten distinguished scientists joined the Editorial Board: Prof. Dr. John E. Kovaleski (University of South Alabama, USA); Prof. Dr. Paul Arciero (Skidmore College; USA); Prof. Conrad P. Earnest (Texas A&M University; USA); Prof. Laura Capranica (University of Rome; Italy); Assist. Prof. Dr. Helmi Chaabene (University of Potsdam; Germany); Dr. Abdul Rashid Aziz (Singapore Sport Institute, Singapore); Dr. Michael O. Harris-Love (Veterans Affairs Medical Center; USA); Dr. James Fisher (Southampton Solent University; UK); Dr. Roland van den Tillaar (Nord University; Norway); and Dr. Cristina Cortis (Society and Health University of Cassino e Lazio Meridionale; Italy), for a total of 72 editorial board members, eight advisory board members, and the editor-in-chief.

Journal of Functional Morphology and Kinesiology confirmed its status as an international journal by being present as a media partner, an exhibitor, or a sponsor at national and international meetings, among which were the following:

- 2018 ORS (Orthopedic Research Society) Annual Meeting, 10–13 March 2018, at the Hyatt Regency New Orleans, USA, (sponsored journal poster);
- The 23nd Annual Congress of the ECSS, Dublin, Ireland; 4–7 July 2018 (sponsored journal poster in coffee break);
- ISBS 2018, Auckland, New Zealand; 10–14 September 2018 (banner advertisement in newsletter);
- International Conference on Kinesiology and Exercise Sciences, Athens, Greece; 30–31 July 2018 (keynote speaker);
- 6th International Congress on Sport Sciences Research and Technology Support—icSPORTS 2018, Seville, Spain; 20–21 September 2018 (set up conference special issue);
- 72° Italian National Congress of SIAI (Società Italiana di Anatomia e Istologia), Parma (Italy); 20–22 September 2018 (journal flyer distribution);
- X° Italian National Congress of SISMES (Società Italiana delle Scienze Motorie e Sportive), Messina (Italy); 05–07 October 2018 (journal flyer distribution).

For the first time this year we recognized the 2018 JFMK Travel Award. This award (600 CHF) is granted to Dr. Gavin Lenton, a postdoctoral research fellow under the supervision of Prof. David Lloyd from Griffith University. Dr. Lenton’s research focuses on musculoskeletal biomechanics. He will present his work at the 2018 World Congress of Biomechanics, which will be held on 8 July 2018, in Dublin, Ireland. It was a very difficult decision with such high-quality applications for the award, and we would like to thank all the applicants in various fields of study for their participation and all of the Award Committee members for their evaluation of the abundant excellent applications. We congratulate Dr. Gavin Lenton on his accomplishment. I take this opportunity to thank again the Evaluation Committee.

## 2. Looking Forward to 2019 

In 2019, we shall continue our efforts to improve the journal through further growth and increased visibility. 

In order to achieve this target and lay a strong foundation for publications in 2019 and application for indexing, we have made the following plans:

- Follow up the planned papers from editorial board members;
- Contact international conferences recommended by the editor-in-chief or by editorial board members and try to establish media partnerships with them to make JFMK increasingly well-known among scholars;
- Communicate with editorial board members regularly and ask for their help and suggestions for journal development;
- Post high-quality papers through social media (e.g., LinkedIn, Twitter, and Facebook) and increase online readership;
- Reduce the processing time of each manuscript;
- Try to have publications indexed by the Emerging Sources Citation Index (Web of Science) and by EMBASE (Elsevier);
- Try to be included in the SCImago Journal Rank in the kinesiology-related section;
- Accomplish, for our authors, the best JFMK paper award and the JFMK travel grant award;
- Garner, for the sake of journal promotion, support from sponsors for our editors to participate in, and disseminate our journal to, international conferences.

We hope that you share our enthusiasm for this new journal and we look forward to working with you to make JFMK a leader in its field. Your contributions are vital for the success of this new journal. We look forward to receiving your contributions (papers, reviews, etc.) and proposals for special issues are always welcome.

It is my pleasure to end this editorial by wishing you a healthy and prosperous new year. This is also the opportunity for me to warmly thank, for their confidence, the following: our authors, readers, and reviewers, as well as our editorial advisors, eminent scientists in these fields that with their experience and important suggestions, guide us in this great enterprise; our excellent editorial board members whose depth of experience covers a very broad spectrum on different disciplines related to morphology and kinesiology arenas; the managing editor Olivia Yu for her huge support, the publishing manager Martyn Rittman, the assistant managing editor Molly Lu, and assistant editor Sydney Tang, who day after day, thanks to their valuable contributions, ensure the growth of this journal; and, finally, all members of our teams in Basel, Barcelona, Beijing, Belgrade, and Wuhan, as well as our sponsors.
